# Bacterium-like particles derived from probiotics: progress, challenges and prospects

**DOI:** 10.3389/fimmu.2023.1263586

**Published:** 2023-10-06

**Authors:** Xinyao Zhou, Mingchun Gao, Xinqi De, Tong Sun, Zhikun Bai, Jilong Luo, Fang Wang, Junwei Ge

**Affiliations:** ^1^ College of Veterinary Medicine, Northeast Agricultural University, Harbin, China; ^2^ School of Basic Medical Sciences, Youjiang Medical University for Nationalities, Baise, China; ^3^ State Key Laboratory of Veterinary Biotechnology, Harbin Veterinary Research Institute, The Chinese Academy of Agricultural Sciences, Harbin, China; ^4^ Heilongjiang Provincial Key Laboratory of Zoonosis, Harbin, China

**Keywords:** lactic acid bacteria, bacterium-like particles, antigen delivery, carrier-adjuvant, vaccine

## Abstract

Bacterium-like particles (BLPs) are hollow peptidoglycan particles obtained from food-grade *Lactococcus lactis* inactivated by hot acid. With the advantage of easy preparation, high safety, great stability, high loading capacity, and high mucosal delivery efficiency, BLPs can load and display proteins on the surface with the help of protein anchor (PA), making BLPs a proper delivery system. Owning to these features, BLPs are widely used in the development of adjuvants, vaccine carriers, virus/antigens purification, and enzyme immobilization. This review has attempted to gather a full understanding of the technical composition, characteristics, applications. The mechanism by which BLPs induces superior adaptive immune responses is also discussed. Besides, this review tracked the latest developments in the field of BLPs, including *Lactobacillus*-derived BLPs and novel anchors. Finally, the main limitations and proposed breakthrough points to further enhance the immunogenicity of BLPs vaccines were discussed, providing directions for future research. We hope that further developments in the field of antigen delivery of subunit vaccines or others will benefit from BLPs.

## 1 Introduction

Lactic acid bacteria (LAB) encompass a collection of Gram-positive bacterial species that engage in the process of carbohydrate fermentation, resulting in the production of lactic acid. Notable members of this group include *Lactobacillus*, *Lactococcus*, *Streptococcus*, *Pediococcus*, *Enterococcus*, *Leuconostoc* and various others (1). Traditionally, LAB are utilized in the process of fermentation and preservation of various dairy, meat and vegetable products (2). Nowadays, a large number of LAB have obtained generally regarded as safe (GRAS) status designation from the American Food and Drug Administration (FDA), and widely take safe part in various fields in humans and animals (3). More importantly, many LAB are probiotic. LAB and their active metabolites exhibit many properties such as anti-cancer (4), anti-bacterial (5, 6), anti-viral effects (7), reduce intestinal inflammation (8–10), allergies (11) and serum cholesterol (12). Given these valuable merits, LAB have been chosen to develop as safe platforms for the surface display of heterologous proteins for medical applications (13).

A novel LAB surface display technology based on bacterium-like particles (BLPs), also termed gram-positive enhancer matrix (GEM), was first proposed and developed by Bosma et al. (14) BLPs are LAB killed by hot acid, overcoming the safety concerns caused by the need for genetic modification of live LAB carriers (15). This inexpensive, biosafe, effective, and easy-to-handle heterologous protein display system has been applied in vaccine immunization, antigen purification, enzyme immobilization, etc.

So far, BLPs have participated in more than 40 different varieties of bacterial, viral and parasitic vaccine designs, and provided 100% protection against influenza virus (16), Newcastle disease virus (17), *Streptococcus pneumoniae (* 18), and *Plasmodium berghei (* 19). Furthermore, the first phase I clinical trial with a BLPs-based Flu vaccine in humans was published in 2013 (20), subsequently, the respiratory syncytial BLPs vaccine also achieved good results in phase I clinical trial in 2019 (21). As the field step to human clinical trials, it is time to consolidate what has been achieved so far, identify knowledge gaps, and discuss the future possibility of BLPs.

In order to comprehensively summarize the research progress of BLPs, this paper reviews the literature in recent years. At present, Kees Leenhouts reviewed BLPs-based vaccine delivery (22) and summarized the effect of BLPs on enhancing mucosal immunity (20). Wang et al. reviewed the development of BLPs as a novel surface display technology and its application (23). However, there was not any comprehensive review on the preparation, advantages, application, and mechanisms of BLPs as tools. In this review, we will comprehensively summarize technical composition, characteristics, application and immune enhancement mechanism of BLPs, and discuss the limitations and future development directions. As a subunit vaccine carrier and adjuvant, BLP solves the problem of poor immunogenicity of subunit vaccines. We believe that BLP will have great application potential in more biomedical fields due to its favorable properties.

## 2 Systematic composition

**Figure 1:**
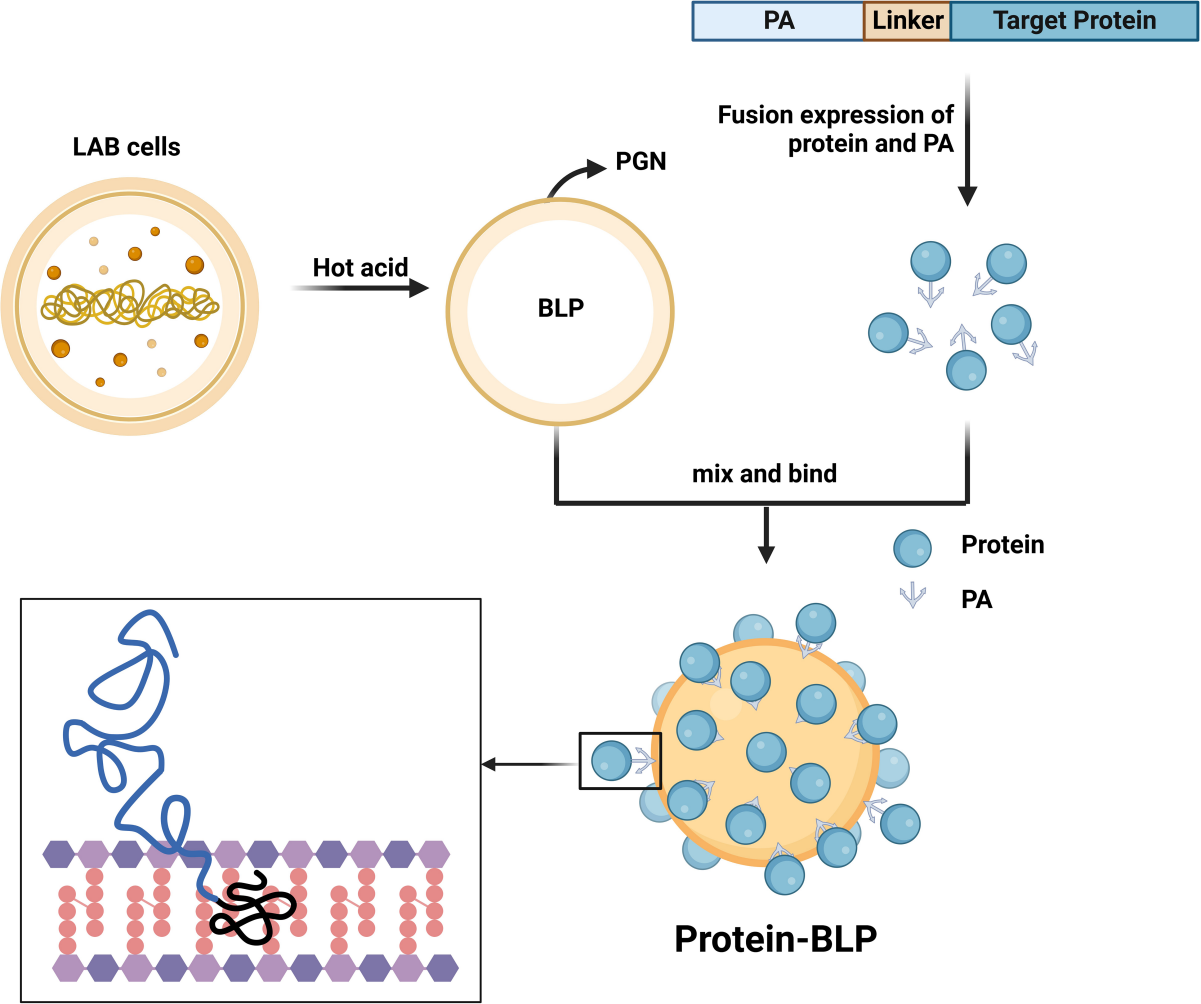
Preparation process of BLPs display system. LAB after hot acid treatment left only the PGN shell which is BLP. The protein and PA are fused and expressed in a suitable host, and a linker can be added to enhance flexibility. Mixing the protein-PA solution with BLP produces a strong and stable non-covalent binding, allowing the BLP to be completely covered with protein on the surface. Created with BioRender.com.

The systematic composition of BLPs surface display system consists of three parts: BLPs (scaffold unit), protein anchor (PA) (anchor unit) and recombinant target protein (active unit) (**Figure 1**). The protocol for preparation of BLPs surface display system were showed in **Supplementary Text 1**.

### 2.1 BLPs preparation

Bosma et al. first described a simple and rapid method for BLPs preparation, which is still in use to date: *Lactococcus lactis* are boiled in hot acid for 30 minutes. This procedure deprives the entire intracellular contents of bacterial cells including proteins, nucleic acids and lipophosphatidic acids and extracellular macromolecules. And the degradation products and acid will be removed by washing with buffer three times (14). It has been found that hot HCl, TCA or H_2_SO_4_ treatment led to successful BLPs production, and the acid concentration of 10% is the best (24). After treatment, the resulting hollow particles essentially are *L. lactis* cell wall peptidoglycan (PGN) shell which retain their original shape and size (1–2 μm) after treatment. This method is generally applicable to gram-positive bacteria, not only for *L. lactis*. Apart from *L. lactis*, the peptidoglycan backbones of *Lactobacillus plantarum*, *Lactobacillus salivarius*, *Lactobacillus rhamnosus* and *Corynebacterium pseudodiphtheriticum* are also resistant to hot acid treatment and can also be successfully prepared into BLPs (25–27). More details regarding *Lactobacillus*-derived BLPs are provided in the following “Advances in *Lactobacillus*-derived BLPs development” section.

### 2.2 PA

PA is currently the most widely used anchor in the BLPs system. With the bridging of PA, BLPs can be used as display carriers for exogenous proteins. PA derives from the peptidoglycan-binding domain of *L. lactis* cell-wall hydrolase AcmA which consists of 437 amino acids and contains three domains: N-terminal signal sequence region, central active region, and C-terminal domain referred to as PA. PA contains three repeating lysine motifs (LysM), which are composed of about 45 amino acids separated by spacer sequences. LysM is a very ubiquitous cell wall-binding domain (CWBD), primarily present in cell wall hydrolases for bacteria (28).Bateman and Bycroft analyzed the three-dimensional structure of LysM. The results of nuclear magnetic resonance (NMR) showed that the LysM domain derived from membrane-bound lytic murein transglycosylase D (MltD) in *Escherichia coli* contains two LysMs which has a βααβ secondary structure (29). Strong binding of LysM to bacteria cell wall has been previously reported in a patent (30). The specific binding of the LysM motif to the peptidoglycan backbone is by non-covalent bonding, and the binding ability is so strong that anchored protein can only be dissociated by SDS or 8M LiCl treatment. In this case, the binding of the protein of interest to BLPs can be achieved by fusing the protein with PA.

### 2.3 Target protein

At present, the essence of molecules displayed on the BLPs surface are proteins or peptides. Most proteins displayed on the surface of BLPs are pathogenicity-related proteins of bacteria, viruses and parasites, which are used to improve the immune effect of subunit vaccines. Protein-PA fusions have been expressed successfully by prokaryotic hosts (*E. coil* and *L. lactis*) and some eukaryotic hosts (insect, yeast, tobacco, HEK, and CHO cells). Simple incubating target protein fused with PA and BLPs can realize the display of exogenous protein. The addition of protein does not seem to affect the high affinity of PA for BLPs. Notably, both in the biophysical properties (31, 32) and biological functions, various protein molecules displayed on the surface of BLPs have shown promising activity and function.

### 2.4 Linker

Protein linkers are crucial in enhancing binding capacity by facilitating mobility and flexibility in the fusion protein. This enables the protein to achieve an ideal orientation within the peptidoglycan structure (33). In terms of vaccine development, it has been shown that the use of flexible linker peptides “(Gly-Gly-Ser-Gly) × 2” between the PA and antigenic protein RBD could enhance the binding ability of the PA thereby improve the immunogenicity of the RBD in the MERS-CoV BLPs mucosal vaccine (34). In their subsequent work, this linker was also inserted between the PA and antigenic protein Sudan virus (SUDV) glycoprotein (eGP). The intramuscular administration to mice of adjuvanted eGP-BLPs elicited high specific IgG and neutralizing antibody titers. Likewise, another study has also applied a flexible linker “(Gly-Gly-Gly-Gly-Ser) × 3” between the PA and antigenic protein gp120 to the design of HIV BLPs vaccine which achieved good immune effects (35, 36).

## 3 Characteristics of BLPs

### 3.1 Structure and composition of BLPs

**Figure 2:**
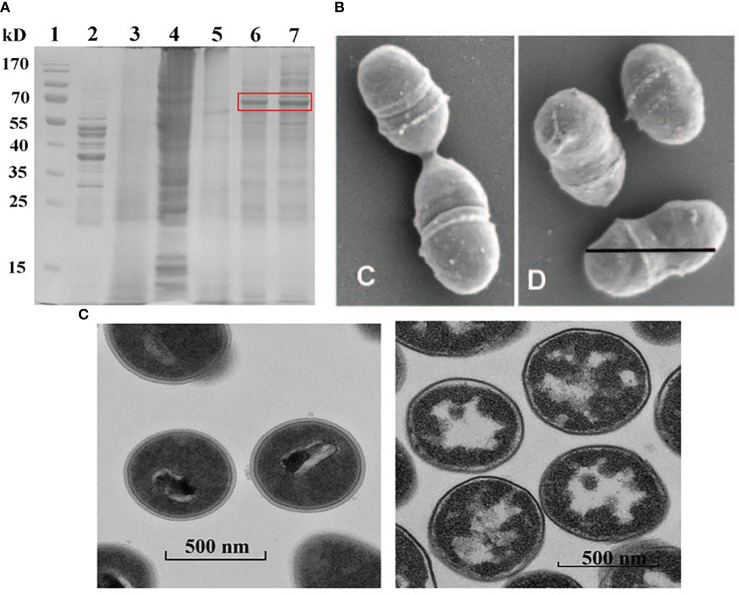
Characterization of BLPs. SDS-PAGE analyses of PA fusion protein and BLP particles **(A)** Reprinted with permission from ref (38). Copyright 2022. Frontiers. lane 1 is protein marker, lane 2 is *L. lactis* MG1363, lane 3 is BLP particles. Scanning electron microscopic analysis of *L. lactis* cells (Left) and BLP particles (Right) **(B)** Reprinted with permission from ref (14). Copyright 2006. ASM journals. Transmission electron microscopy analysis of *L. lactis* cells (Left) and BLP particles (Right) **(C)** Reprinted with permission from ref (17). Copyright 2021. Elsevier.

Experimentally, more than 97.5% of the TCA treated staphylococcus aureus cell wall is N-Acetylglucosamine (the main component of peptidoglycan) (37). In the study of the structure and composition of BLPs, SDS-PAGE analysis denoted that *L. lactis* MG1363 contained its own protein (**Figure 2A**, lane 2), and after 10% TCA treatment, BLPs contained almost no protein components (**Figure 2A**, lane 3) (38). As shown in the Scanning electron microscopic (**Figure 2B**) (14) and Transmission electron microscopy (**Figure 2C**) (17) images, BLPs retain the original bacterial size and shape, but the cellular contents are partially degraded and the peptidoglycan backbone is exposed.

### 3.2 No safety concerns

Ever since Hansson et al. pioneered the display of proteins on the surface of *Staphylococcus xylosus*, numerous proteins have been successfully exhibited on living bacterial surfaces. However, in all instances, the host bacteria are genetically modified organisms (GMOs), potentially carrying foreign and altered genes. This raises concerns about environmental dispersion, horizontal gene transfer to other bacteria, and associated safety risks (14). In comparison, BLPs are prepared from non-pathogenic food-grade LAB from a variety of sources. Heat acid-treated BLPs are non-living, non-genetically modified organisms and do not contain any nucleic acid material, minimizing the risk of transmission of recombinant DNA to the environment. In a preclinical GLP toxicity trial, BLPs were administered intranasally to rabbits, and no adverse events were reported (39). Furthermore, the safety of an intranasal influenza vaccine prepared by mixing seasonal trivalent inactivated influenza vaccine with BLPs has been assessed in humans in a phase I clinical trial, resulting in no adverse events reported (20).

### 3.3 Great binding stability

BLPs in the absence of extracellular components bind to PA strongly on the surface, thus, loaded BLPs were stable storing in PBS or as a freeze-dried powder at room temperature, 4°C, and −80°C, and no loss or degradation of the PA fusions were observed (14). In a representative example, *Yersinia pestis* V immunogen was used to determine the binding force between V-PA and BLPs in AFM-based single-molecule force measurement, and the binding force at physiological pH was about 51.33 ± 5.78 pN which is notably larger than it between HIV gp120 and CD4 (about 42.25 ± 34.14 pN) (40).

Some studies have shown that the number of LysM domain in the anchor effect the stability of BLPs surface display system. As Steen et al. demonstrated, fusion protein with three LysM domains from AcmA has optimal peptidoglycan binding properties and biological activities (41). Indeed, PA with three repeats of LysM is by far the most widely used protein anchor for BLPs. In contrast, one study demonstrated that fusion proteins containing two LysM domains exhibit significantly enhanced binding activity towards BLPs compared to other numbers of domains (14). In a recent study, the binding stability of Venus-LysM proteins to BLPs at different NaCl, urea concentrations and at different temperatures and pH was tested. The result showed that under unfavorable conditions (high concentrations of urea and NaCl and low temperature), the stability of Venus-Acglu-BLPs (containing five repeats of LysM motif) was stronger than that of Venus-Pgb-BLPs (containing one LysM motif), but at different pH (4, 7.4 and 9), there was no statistical difference between them. This result indicated that the higher the number of LysM motifs, the stronger the resistance of Venus-LysM-BLPs to adverse conditions at equimolar concentrations. However, the study also showed that five LysM motifs from Acglu did not exhibit a greater binding capacity to BLPs than one LysM motif in Pgb at the same micromolar concentration (42). Therefore, we deduced that rather than the optimal number of LysM domains, it seems that the binding stability depends more on the protein from which the anchor is derived.

### 3.4 High loading capacity

Compared with live *L. lactis* surface display system, BLPs with their peptidoglycan binding sites fully exposed have a higher PA display density which distribute over the entire surface of BLPs (43). As determined by Bosma et al., the maximum PA binding capacity is 10^6^ molecules per BLP cell compares favorably with the surface density of PA on BLPs in *Staphylococcus carnosus* and *Bacillus subtilis* spore surface display platform with 2–3 orders of magnitude enhancement, respectively (14). The single-molecule sensitive NSOM/QD based dipole mission fluorescence imaging system was employed to analyze binding density, and based on a conservative estimate, there were at least ~3000 QD-bound V-PA immunogen molecules on a single bacterium-like particles. Not only that, the density of V-PA immunogen was ~1492 molecules/mm^2^ which is significantly higher than the density of ~866 CD4 molecules/mm^2^ on a CD4^+^ T-cell (40). High loading capacity makes BLPs surface system an excellent platform for the large-amount and high-density loading of proteins.

### 3.5 Induction of both systemic and mucosal immunity

The whole mucosal immune system is interconnected, indicating the vaccination of the local mucosa activates the whole mucosal system, producing antibodies in remote mucosal areas other than the site of vaccination (35). BLPs retain the size of live *L. lactis*, of about 1 μm, suitable for uptake by M cells located in Peyer’s patches located in gut-associated lymphoid tissue (GALT), which together with the nasal-associated lymphoid tissue (NALT) form a typical mucosa immune system (CMIS) (14). Secondly, mucosal immunization, which mimics the natural way pathogens invade the organism, activates mucosal immunity (sIgA) and induces a systemic immune response (IgG). In addition, this needle-free immunization reduces immune side effects and is suitable for mass vaccination and multiple immunizations.

### 3.6 Efficient simultaneous display of multiple antigens

Non-antigen restricted binding of protein-PA to BLP makes it possible for multiple antigenic proteins to be simultaneously anchored to the same BLP cell. Two B-cell antigenic epitopes of *Plasmodium berghei* malaria circumsporozoite protein were simultaneously displayed on BLPs resulting in significant levels of IgG antibodies in vaccinated mice intranasally (19). The trivalent and bivalent vaccine based on three *Streptococcus pneumoniae* proteins IgA1p, SlrA and PpmA provided good protection and reduced bacterial load in mice with no antigen antagonism was observed. Moreover, Moreover, when the three antigens were prepared as a bivalent vaccination or a trivalent vaccine with 1.5 times less of this antigen, the IgG antibodies generated against that antigen were equal (44). Additionally, two forms of bivalent vaccine: tHA^H5N6 +H9N2^ BLPs and a mixture of tHA^H5N6^ BLPs and tHA^H9N2^ BLPs both led to potent immune reactions without additional adjuvants (45).

## 4 Applications of BLPs in biomedicine

**Figure 3:**
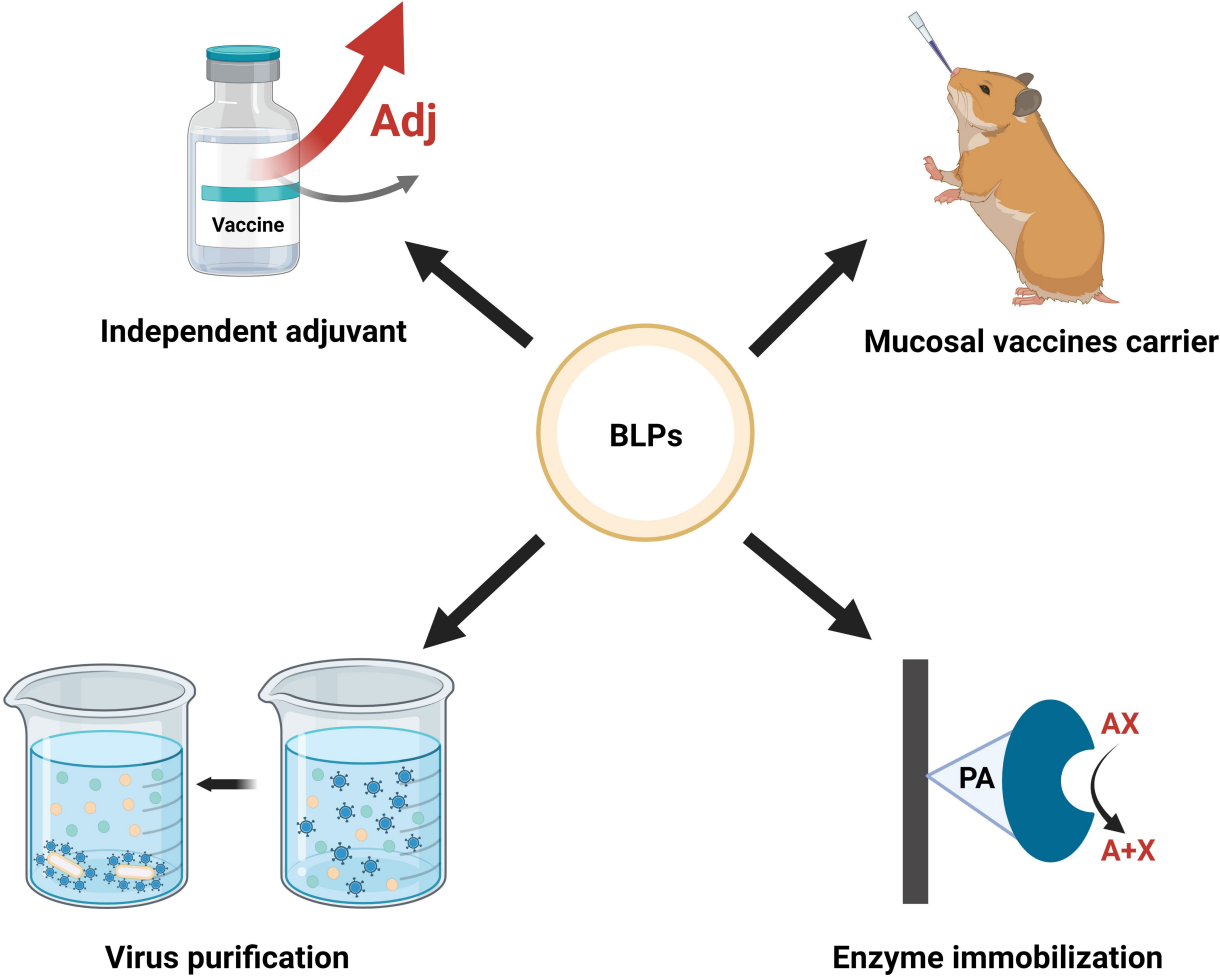
Application of BLPs display system. Created with BioRender.com.

BLPs are widely used in the development of vaccine carriers, adjuvants, virus/antigens purification, and enzyme immobilization or biocatalyst targets. As an illustration, applications of BLPs in biomedicine are shown in **Figure 3**, immune effects as vaccine carrier/adjuvant are shown in **Supplementary Table 1**.

### 4.1 BLPs-based delivery system for vaccination

Vaccination is one of the most effective preventative measures against pathogenic infection. According to World Health Organization estimates, it is determined that vaccines prevent 3.5–5 million deaths annually (https://www.who.int/health-topics/vaccines-and-immunization#tab=tab_1). And in the first year after the COVID-19 vaccination, it saved nearly 14.4 million lives around the world (46). While for some diseases, there is no effective vaccine available (47). Therefore, novel vaccines combined safety and immunogenicity is desperately needed. Using bacteria-based carrier as novel vaccine strategies, people set their sights on BLPs.

#### 4.1.1 Bacterial vaccines

The oral administration of bivalent and trivalent BLPs vaccines prepared from three pneumococcal proteins, IgA1p, PpmA and SlrA, induced specific serum IgG production in mice, and provided up to 70% protection against lethal challenge with reduction of bacterial counts in blood nose and lungs (44). Wu Yongge’s team has also applied BLPs to *Streptococcus pneumoniae* vaccines. Plym2 is a highly detoxified mutant of pneumococcal haemolysin (Ply). Intranasal inoculation of BLPs carrying Plym2 induced increased levels of IgG antibodies in mice serum and mucosal IgA antibodies in lung lavage fluid, and the neutralizing antibody activity was significantly higher than that of intramuscular injection of Plym2 alone (48). Another study showed that PapA3, a fusion protein of the three repeats of *Streptococcus pneumoniae* protein PspA, was displayed on the surface of BLPs and used to immunize mice intranasally. After the homologous heterologous *Streptococcus pneumoniae* challenge, PspA3-BLP provided 100% protection in mice, outperforming the commercial 23-valent polysaccharide vaccine (50%) (49).

A study was conducted to evaluate the immune effect of BLPs loaded with *Shigella* invasion plasmid antigens IpaB and IpaD separately in young rats after intranasal immunization, resulting in an increased level of specific serum antibodies and a 90% and 44% protection against cross-infection with *Shigella fowleri* and *Shigella sonnei*, respectively (50). Previous studies have reported that the *Yersinia pestis* LcrV-displayed BLPs activated the neonatal immune system through mucosal inoculation. Intranasal inoculation of LcrV-BLPs activated a robust mucosal and systemic immune response in newborn mice: serum-specific IgG remained high at the time point of the last assay (postnatal day 77); large numbers of IgA and IgG LcrV-specific antibody-secreting cells (ASCs) were present in the NALT; increased levels of IL-2, IL-12, IL-6, TNF-α, and IL-10 were detected in the NALT, and IL-10 levels were detected in NALT. Finally, LcrV-GEM provided 100% protection against *Y. pestis* lethal challenge, while LcrV without GEM only provided mice 20% protection (18).

Very often, *Helicobacter pylori* is considered a non-invasive pathogen residing in the extracellular mucus layer, therefore, prevention and treatment of *H. pylori* by mucosal route is an ideal approach. Xing’s team combined cholera toxin B subunit and multiple copies of the B cell epitopes as well as T cell epitopes from the *H. pylori* urease A and B subunits for multi-epitope vaccine CTB-UE (CUE), consequently were utilized to treat *H. pylori* infection. Oral administration of CUE reduced urease activity, bacterial colonization and levels of gastric inflammation in a mice model of *H. pylori* infection through the production of mucosal antibodies and cytokine (IFN-γ and IL-17) (51). Subsequently, Xing’s team found that the protective effect of pre-orally immunized CUE-BLPs in mice against *H. pylori* was associated with Th17 innate immune regulation in the stomach and MLN, and this protective effect was independent of systemic antibodies, but is manifested by markedly elevated levels of immune response cytokines including neutrophil chemokine CXCL1-2, neutrophils, antimicrobial peptide S100A8 and MUC1 (52).

#### 4.1.2 Viral vaccines

A BLPs-based HIV-1 vaccine displays the HIV-1 glycoprotein gp120-MTQ. gp120-MTQ bound to BLPs, rather than mixed BLPs, can elicit systemic and mucosal response in mice and guinea pigs by intranasal immunization. Furthermore, nasal rinses of guinea pigs showed neutralizing activity against HIV-1 class I pseudovirus (35). The team evaluated the effectiveness of the vaccine by immunizing rhesus macaques in combination. In terms of mucosal immunity and systemic T cell immunity, BLPs-based vaccine showed significant advantages over BLPs alone (53). BLPs-RSVF, which displayed the respiratory syncytial virus (RSV) F protein on its surface, induced high levels of nasal sIgA and serum neutralizing antibodies than inactivated vaccines by immunizing BALB/c mice or cotton mice nasally, and significantly reduced lung viral load after a strong viral challenge. These promising results made it a safe and effective vaccine candidate against RSV and currently licensed for UK drug clinical trials (20). Intranasal immunization of mice BLPs vaccine demonstrating the MERS-CoV receptor binding domain (RBD) increased systemic, cellular and mucosal immune responses, particularly in the intestine (34). BLPs carrying Rift Valley fever virus (RVFV) Gn head protein induced both humoral and cellular immunity in mice, and antibodies produced by immunized mice showed effective neutralizing activity against RVFV pseudoviruses (38). BLPs vaccine constructed from the E envelope protein of tick-borne encephalitis virus (TBEV) were co-immunized with ISA 201VG, Poly(I:C) adjuvant in mice, and the cellular and humoral immune responses produced were better than those immunized with the adjuvant alone. Surprisingly, high levels of serum specific IgG titers 10^6^ are maintained 6 months after the second immunization (54). Moreover, iridovirus major capsid protein (MCP) with coiled-coil domain of coronin 1 (ccCor1) was attached to the surface of BLPs. Intraperitoneal injection in mice which induced to produce high levels of specific IgG antibodies (55).

#### 4.1.3 Parasitic vaccines

Without the use of additional adjuvants, oral immunization with BLPs vaccine exhibiting the protective protein MSA2 of *Plasmodium falciparum* cleavers resulted in increased serum antibody titers in rabbits. In addition to this, the anti-vector effect of the MSA2 BLPs vaccine was greatly reduced compared to that of the recombinant live LAB vaccine (56). *Plasmodium berghei* circumsporozoite protein (PbCSP) peptides loaded BLPs induced high specific IgG levels and number of IFN- γ spots and complete protection against malaria in mice (19).

### 4.2 BLPs for vaccine adjuvants

Acting as adjuvants, free-standing BLPs can be mixed simply with existing vaccines or purified antigens to enhance immunogenicity. In particular, the effect of BLP on enhancing the immunity of influenza vaccines has been intensively studied by Leenhouts et al. Adjuvantation of IN-administered influenza monovalent split vaccine of strain A/Beijing/262/95 (H1N1) with BLPs effectively enhanced serum IgG and HI levels and sIgA titers in nose, lung and vaginal in mice resulting in a 100% protection against homologous and heterologous influenza infection with reduction of viral titer in lungs (16). Oral immunization with monovalent subunit vaccine of strain A/Hiroshima (H3N2) mixing BLPs elicited not only systemic and local antibody responses but also a more balanced Th1/Th2 response compared to inactivated influenza vaccines added with alum (57). The administration of combined BLPs and seasonal HA (A/Wisconsin/67/2005 [H3N2]) resulted in notably elevated systemic immune responses and a more balanced Th1/Th2-type immune response compared to the administration of HA alone via the intramuscular route. The addition of BLPs HA saved 25–125 times of the antigen dose and resulted in similar HI titers (58). What’s more, just one boost is required for i.n. BLPs adjuvanted influenza monovalent subunit vaccine to reach an equal level of HI titers in serum to the conventional i.m. route. Nasal immunization with a subunit vaccine mixed with BLPs led to a decrease in the quantity of IL-4-producing cells, while significantly increasing the quantity of IFN-γ-producing cells, indicating a shift of the immune response from a balanced Th1/Th2 to a predominant Th1-type response (39). Nasal vaccination with BLPs adjuvanted split influenza vaccine of influenza A strain (A/Sydney/5/97[H3N2]) induced IAV-specific IgG2c antibody production and Th1/Th17 immunological responses (59). The safety and immunogenicity evaluation of BLPs-based vaccines in humans achieved good results in FluGEM-a phase I clinical trial (20). A/turkey/Turkey/1/2005 (H5N1) virus fatal viral challenge by the combination intranasal/intratracheal route is entirely prevented by passive inhalation of dry powder influenza vaccine formulations, and no virus was detected in choanal or cloacal swabs of vaccinated chickens (60).

Admixing low doses of BLPs with a live H120 IBV vaccine via ocular administration to chickens demonstrated the ability of rapidly boosting higher specific serum IgG titer levels, even at two weeks after hatching (61). BLPs mixed with inactivation of NDV antigens induced high levels of specific serum IgG and mucosal IgA titers. Intranasal vaccination of chickens results in serum HI titers of up to 2^8^, providing a complete protection against a challenge with a fatal dose of virulent NDV without any illness symptoms (17). Oral immunization with commercial live rotavirus vaccine with BLPs derived from immunomodulatory *Lactobacillus rhamnosus* resulted in enhanced levels of specific serum IgG and IgA, increased numbers of CD24^+^B220^+^ B and CD4^+^ T cells in Peyer’s patches and spleen as well as augmented production of IFN-γ (25). Similarly, oral immunization with hepatitis E virus capsid protein (ORF2) with BLPs also achieved a satisfactory immunity effect (26).

### 4.3 BLPs-derived virus/proteins purification tools

Vaccines for the prevention or treatment of viral diseases are mainly based on attenuated or inactivated viruses and recombinant viral proteins that are produced within different host cells. However, in the production process of viruses, downstream purification processing has always been a challenge due to the different structures and properties of different viruses (62). An optimal clarification process should effectively eliminate cellular debris and sizable aggregates, which may arise from either the production or processing stages (63). The usual methods of virus purification are ultracentrifugation precipitation (density gradient), ultrafiltration and size exclusion chromatography (molecular size), ion exchange chromatography (charge) and affinity chromatography (specific binding). However, these methods require for significant time and labor input, and the purity and yield of purification cannot be guaranteed. There are many limitations in applying it to vaccine production, especially heterologous substances such as foreign proteins, nucleic acids, lipids, and carbohydrates in the culture medium can easily cause immunosuppression and immune side reactions.

The addition of cell-free culture medium that contains a PA fusion to BLPs leads to a highly effective, strong, and specific surface binding of fusion protein, eliminating the requirement for supplementary purification measures. A single-step centrifugal procedure can be employed to eliminate the recombinant producer cells (14). GRFT, a lectin from the marine red alga *Griftsia sp*, can tightly bind to glycoproteins on the surface of many enveloped viruses. After incubation of PA-GRFT with Porcine reproductive and respiratory syndrome virus (PRRSV) and BLPs, one-step low-speed centrifugation was employed in order to isolate purified PRRSV. Further experimental results demonstrated that the PA-GRFT partnership could guide PRRSV to the BLPs surface, yielding favorable results in terms of efficiency (64). Additional research using this process to purify additional enveloped animal viruses may result in a brand-new, broadly applicable technique for viral purification (65). Another typical study connected foot-and-mouth disease virus (FMDV)-specific nanobody to BLPs through PA, named GEM-PA-nanotrap, was used to FMDV purification from cellular lysates. The recovery rate of FMDV exceeded 99% and the efficiency of removing nonviral proteins was approximately 98.3% with no effect on activity (66). This method has also been applied to purification of porcine circovirus type 2 (PCV2). They designed a bifunctional recombinant protein linker by fusing the variable heavy chain domain (VHH) antibody directed against PCV2 with PA. 100 mL of PCV2-containing culture supernatant (viral titer: 10^6.5^ TCID50 mL^-1^) could be purified using 1 unit (2.5×10^9^ particles) of BLPs carrying 80 μg protein linker with a recovery rate up to 99.6%. Based on the decreased total protein content after purification, this technique has an impurity removal effectiveness of about 98%. Moreover, the results of *in vivo* experimentation demonstrated that piglets that were immunized with purified PCV2 exhibited robust immune responses, effectively protecting them against PCV2 infection (67).

### 4.4 BLPs-derived enzyme immobilization or biocatalyst targets

In large-scale industrial and biotechnological applications, enzymes play a growing role as effective and versatile biocatalysts. Enzyme-based catalytic processes have many advantages over traditional synthetic routes (64). Many industrially relevant enzymes are produced by microorganisms under extreme conditions, which will face two problems: enzymes still need to be purified and ensure the stability of the enzyme. Immobilizing enzymes on different types of supports is a highly appealing concept for addressing these limitations (68, 69). Appropriate immobilization systems can improve the stability of enzymes under harsh reaction conditions such as extreme pH, high temperature or the presence of organic solvents, making it easier to separate from the reaction medium. Also, certain immobilization procedures allow simultaneous purification and immobilization of enzymes from crude extracts (70).

Mediated by PA, *B. licheniformis* α-amylase (AmyL) and *E. coli* β-lactamase can be immobilized on the surface of BLPs in controlled ratios with no need for chemical treatments or extensive purification steps (14). Subtilisin QK-2 surface displayed onto BLPs was more stable in the simulated gastric juice without a loss of fibrinolytic activity (71). The BLPs could immobilize over 75% of the enzyme activity of lipases with no need to add ions to improve the activity (72).

## 5 The mechanism of BLPs on immune system

**Figure 4:**
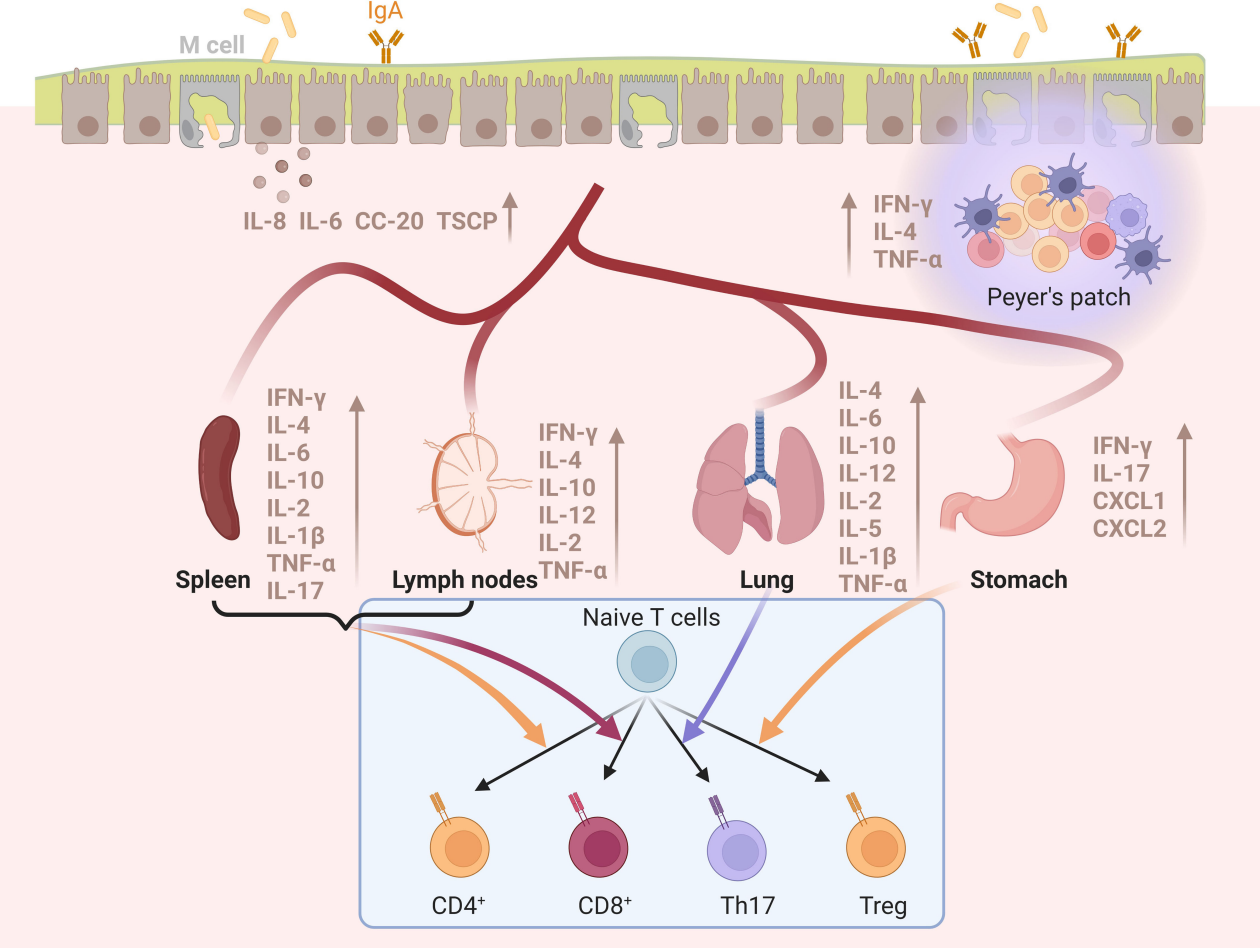
The immune responses of BLPs-based vaccines in different tissues and organs produced by mucosal vaccination. Mucosal immunization of various BLPs-based vaccines can induce the production of various cytokines in the spleen, lymph nodes, lung, stomach, and mucosal tissue, and stimulate the differentiation of native T cells and antibody production. Created with BioRender.com.

The effect of the vaccines listed above has repeatedly shown that vaccines or antigens loaded/mixed with BLPs can induce specific immune responses offering effective protection against pathogens successfully. The mechanism of BLPs vaccines providing high protection will be analyzed from the three aspects of BLPs-acting cells, receptors and secreted cytokines. Representative sections are shown in **Figure 4**.

### 5.1 Antigen-presenting cells

DCs are the most critical antigen-presenting cells (APCs) with the strongest ability to activate T-naive cells. It has been shown that BLPs activate and promote the maturation of BM-derived CD11c^+^ cells of neonatal and adult mouse *in vitro* exhibiting an increased expression of activation and maturation cell-surface markers (CD80, CD86, CD40 and MHC-II) and secretion of proinflammatory cytokines (IL-12p70, TNF-α, IL-10, IL-6, IFN-γ, and MCP-1) (18). DCs maturation was also observed an *in vivo* study. An increase in CD19^+^ CD40^+^ double-positive cells nodes and CD11c^+^ MHC-II^+^ cells was observed inguinal lymph nodes early in Zika virus E protein-BLPs vaccine immunization (73).

As key executors of innate and adaptive immunity, macrophages play an important role. BLPs uptake in macrophages was observed in a previous study (74), along with the production of TNF-α. Another study showed both RAW264.7 and DC2.4 cells had a strong ability to uptake BLPs displaying CSFV E2 protein (75).

### 5.2 Epithelial cells and M cells

Respiratory mucosal tissues are equipped with diverse antigen uptake and presentation systems, and antigen transport across the nasal epithelial barrier is the first step in the induction of a mucosal immune response upon mucosal vaccination (76). Antigens in the nasal cavity are taken up in three forms: epithelial cells, M cells, and lamina propria DCs (77). Epithelial cells, as a non-professional APC, play a certain role in the uptake and presentation of antigens. By using an *in vitro* human nasal epithelial cell activation assay, it was demonstrated that the activation of *in vitro* human nasal epithelial cells by BLPs leads to the upregulation of IL-6 and IL-8. Additionally, the expression of chemotaxis and activation-related factors of DCs, CCL-20 and TSLP, is also induced by BLPs (78).. M cells are highly specialized epithelial cells with unique structural advantages compared to other epithelial cells, especially the deep invagination of the basolateral membrane to facilitate the encapsulation of lymphocytes (79). The distinctive capabilities of M cells allow for the targeted and effective transport of inhaled or ingested luminal antigens to APCs situated in the M cell pocket or in close proximity to the FAE of MALT. These functions are crucial for the uptake and internalization of antigens, as well as the initiation of immune responses within the mucosal system (79). In order to study the interaction of M cells with BLPs, mice were immunized intranasally with fluorescently labeled BLPs. Staining of M cells in mouse nasal mucosa after 15 min showed efficient uptake and internalization of BLPs by M cells (78). In addition, some lamina propria DCs extend their dendrites through the epithelial layer into the lumen, which can directly uptake antigens in the lumen (80).

### 5.3 T cells

Innate immune cells act as a bridge between innate and adaptive immunity by presenting antigens and specifically activating T cells. It has been shown that DCs pretreated with *Y. pestis* LcrV-attached BLPs stimulated CD4^+^ and CD8^+^ T lymphocytes proliferation significantly *in vitro* and induced an Th1-type response. The cytokines analysis showed that IL-2, IL-12, IL-6, TNF-α, and IL-10 levels increased in the NALT and lung cells produced IL-2, IL-6, IL-5 and IFN-γ under *in vitro* stimulation (18). High proportions of CD4^+^ CD69^+^ T and CD8^+^ CD69^+^ T cells and the production of IFN-γ, IL-6, IL-4, and IL-10 were observed after *in vitro* stimulation of splenocytes from Zika virus E protein-BLPs vaccinated mice (81). BLPs adjuvanted rotavirus vaccine and hepatitis E virus capsid protein (ORF2) both induced increased CD45^+^CD3^+^CD4^+^ T cells and levels of IFN-γ, TNF-α, and IL-4 in Peyer’s patches and spleen (25, 26). Oral immunization with Helicobacter pylori antigen CTB-UE anchored BLPs induced a Th1/Th17 memory response owing to proliferation of Th17 cells in the stomach and MLN. Oral inoculation of single-chain insulin (SCI-59) analog attached BLPs repaired Th1/Th2 imbalance and increased CD4^+^CD25^+^FoxP3^+^ Tregs in the PLN CD4^+^ T cell compartment (82).

### 5.4 B cell

BLPs vaccines can activation of B cells produces plasma cells, which induce high-affinity antibodies and memory cells, thereby protecting the body from pathogen attack. The study found an increased in CD69 B cells from mouse splenocytes (34), IgG ASCs (specific antibody-secreting cells) in the spleen, BM and NALT and IgA ASCs in NALT (18, 50), CD19^+^ CD40^+^ double-positive B cells in inguinal lymph (81) and CD24^+^ B220^+^ cells in Peyer’s patches (25, 26) were observed in previous studies.

### 5.5 TLRs

Innate immune system recognizes antigens through pattern recognition receptors (PRR), of which family of toll-like receptors (TLRs) play important roles. TLRs distributed on a broad spectrum of various immune cell membranes or endosomal membranes recognize corresponding ligands is key to activate the innate immune system. Peptidoglycan, the main component of BLPs, is a ligand of TLR2. TLR2 coupled to TLR1 or TLR6 functions as a heterodimer and recognizes BLPs results in the activation of innate immune cells. It has been shown by Ramirez et al. that BLPs only interacted with HEK293 cells expressing human TLR2 leading to NF-κB activation (18). Studies on BLPs-activated TLR receptors are also being conducted *in vivo*, in one study using BLPs mixed split influenza vaccine. Nasally immunized TLR2KO mice showed decreased serum IgG response and sIgA levels in the nasal and lung lavages and lower numbers of IFN-γ producing T-cells and B-cells both in the local dLN and spleen, compared with wt control mice (59).

Additionally, there is a growing interest to applying BLPs to avian vaccines, to this end, explore the novel mechanism of action of BLPs in birds is needed. Yang et al. found by NF-κB reporter analysis that DF-1 cells (chicken embryo fibroblast cells) recognize BLPs through the combination of chTLR2t1 and chTLR1t1, and this result is consistent with the latest research (83). Furthermore, in HD11 cells (chicken macrophages), BLPs caused NO production and increased IFN-γ, IL-6, IL-1β and iNOS levels (17).

## 6 Advances in *Lactobacillus*-derived BLPs development

Previous works have focused mostly on *L. lactis* BLPs, while recently, considering further improvements in the effect of BLPs, some lactobacillus BLPs have been utilized to develop vaccine adjuvants.

Pinto’s team considered that the immunomodulatory activity of LAB depends on the characteristics of each strain, so that, they isolated two LAB strains with immunomodulatory activity: *L.rhamnosus* CRL1505 and *L. rhamnosus* IBL027 and prepared them into BLPs named IBLP1505 and IBLP027 (25, 26). Compared with mice orally immunized with BLPs from non-immunomodulatory strain L. plantarum CRL1905 adjuvanted live rotavirus vaccine, mice immunized with IBLP027 or IBLP1505 adjuvanted rotavirus vaccine produced higher levels of rotavirus-specific intestinal IgA and serum IgG antibodies and had a greater ability to increase production of TNF-α, IFN-γ, and IL-4 in both Peyer’s patches and spleens (25). The oral administration of IBLP027 and IBLP1505 adjuvanted HEV GT3 capsid protein ORF2 induced mice local and systematic immune responses as opposed to simple antigen, indicating that *Lactobacillus* BLPs have significant adjuvant properties (26). *Lactobacillus salivarius* IBB3154 BLPs, as a carrier to display *Campylobacter spp* hybrid protein rCjaAD, provided chicken efficient protection against oral challenge with *C. jejuni* through *in ovo* route of administration manifested as increased levels of specific intestinal IgA and reduced levels of bacterial colonization (27).

LysM motifs fused fluorescence protein Venus were displayed on the surface of IBLP027. *In vivo*, significantly increased specific BAL IgT and IgA were observed in nasally immunized mice. And, *in vitro*, spleen cells of mice immunized with Venus−IBLPs027 secreted TNF−α, IFN−γ and IL−4 stimulated by Venus (42). A recent study successfully prepared *Lm. fermentum* into BLPs using 5% TCA, showing a stable binding ability (84).

**Figure 5:**
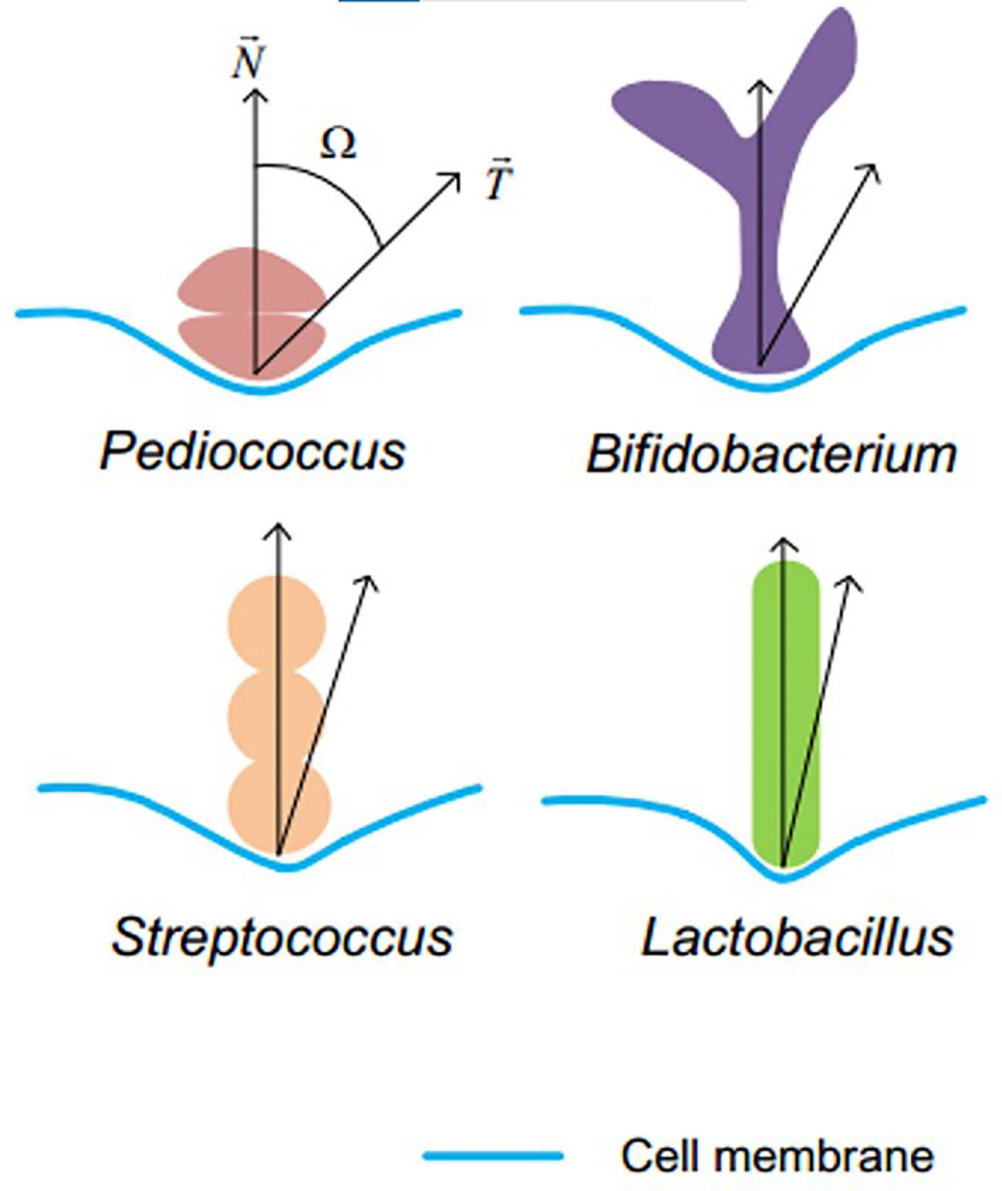
Definition of Ω. *Lactobacillus* (rod shaped) has the minimum Ω angle among the four sets of bacteria Reprinted with permission from ref (73). Copyright 2017. Wiley.

Even if there are few studies on comparison of *Lactobacillus* BLPs and *L. lactis* BLPs in any way as far as we know, but a study of bacteria-induced DC internalization seems to give us a clue. The internalization efficiency of DC is dependent on Ω, defined as the angle between the membrane normal and the line defining the local particle curvature at the cell contact point (**Figure 5**), the smaller Ω, the faster the internalization rate (73) Although the overall process of phagocytosis is the result of a complex interplay between shape and size, particle shape rather than size plays a dominant role in phagocytosis (85). In this case, the *Lactobacillus* BLPs with the smallest Ω value depending only on particle shape might induce the best DC internalization. If *Lactobacillus* BLPs can achieve higher antigen display density or higher immunogenicity, they will be a class of promising alternatives to *L. lactis* BLPs.

## 7 Development of new anchor

Apart from PA, some newly developed connectors are also under study. Two recently described LysM domains from proteins Acglu and Pgb of *Limosilactobacillus fermentum* respectively are fused to fluorescence protein Venus, and both showed the great binding ability to BLPs (42). In addition, given the difficulties of efficient soluble expression of PA fused antigens and the possible side effect of PA addition on antigenicity, an affinity peptide ligand PL23 designed for the classical swine fever virus (CSFV) E2 protein replaced PA and linked the antigen to BLPs by peptide bonds (75). A latest study found that a cell−surface hydrolase (CshA) of *Lactiplantibacillus plantarum* SK156, which does not include any known classical anchoring motifs, can stably bind sfGFP to BLPs (84).

**Figure 6:**
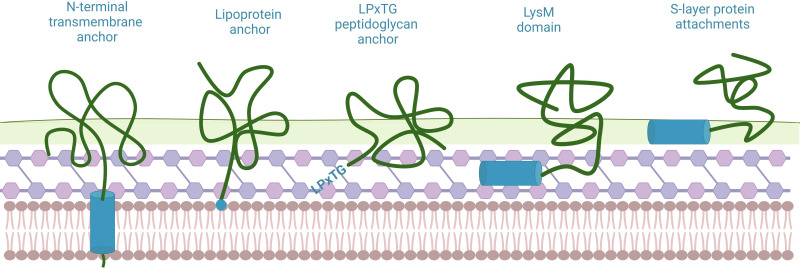
Several classic anchor domains displayed on the surface of LAB. Blue shows anchor domains/motifs, which are coupled to the displayed protein. Created with BioRender.com.

Several classical anchor domains have been found to be capable of binding to LAB, which are divided into covalent binding (N-terminal transmembrane anchor, lipoprotein anchor and LPxTG peptidoglycan anchor) and non-covalent binding (LysM domain and S-layer protein attachments) (86) (**Figure 6**). Among them, the binding site of LPxTG anchor and LysM motif is peptidoglycan, which is the main component of BLPs. Contrary to the widespread application of LysM motif in the field of BLPs, to our knowledge, no studies have applied LPxTG anchor to binding of proteins on the surface of BLPs. Indeed, Peptidoglycan-binding domains (PGBD) are found in a large number of bacterial proteins that interact with the cell wall, such as the cell wall amidases AmiB and AmiC of *E. coli (* 87) and OmpA family lipoprotein of *Pseudomonas aeruginosa (* 88) etc. Even though the application of LysM motif derived from AcmA on the BLPs display system is very mature, we think that more other protein anchors should be candidates for further optimization.

The current research in new anchor is still in its infancy, and the problems exist, for example, existing research are not compared to PA in performance. More research should be carried out, in order to achieve better binding force, stability and practical application effect of BLPs display system, and expand the scope of application.

## 8 Limitation

The activity of proteins displayed on BLPs is related to their native state. The *E. coli* antigen expression system is widely used to produce antigens or epitopes of BLPs vaccine, but the expressed protein may be misfolded and cannot be modified correctly, so the produced protein cannot be guaranteed to be in its native state (89). The limitation of prokaryotic expression system seems to hinder the display of complex and multi-modified proteins on BLPs. Besides, the addition of PA may further increase the difficulty of expressing soluble proteins in *E.coli (* 90).

To address this issue, several trimerization motifs were used to optimize the conformation of BLPs vaccine antigens. Alan et al. retained the F protein in its prefusion conformation by addition of an artificial trimerization motif GCN and mutation of the furin cleavage site. Intranasal immunization of mice and cotton rats with prefusion-like F protein-loaded BLPs induced humoral and mucosal antibody production and reduced pulmonary viral load (91). Trimers of influenza virus HA antigen induced by Coronin trimerization motif (mCor1) and HIV-1 glycoprotein gp120 trimers formed by MTQ were displayed on the BLPs, and both achieved good immune effect (35, 45). Yeast, being a eukaryote, possesses the ability to perform post-translational modifications on proteins, making yeast surface display systems extensively employed in vaccine development (92). Nevertheless, in contrast to the BLPs surface display system, yeast surface display demands a relatively intricate approach. This involves crafting a specialized surface display vector and ensuring the accurate positioning of fusion proteins on the cell surface (93). Consequently, some researchers opt to showcase yeast-expressed proteins on the BLP surface to facilitate the presentation of intricate proteins, such as glycoproteins. Li et al. utilized SpyTag/SpyCatcher system to link the swine fever virus (CSFV) E2 glycoprotein expressed by yeast to PA, realizing the display of glycosylated proteins on BLPs (94). However, purification of glycosylated proteins and optimization of their binding speed to BLPs require further study.

Even though BLPs largely compensates for the poor immunogenicity of subunit vaccines, many BLPs-based vaccines still require multiple immunizations and vaccine adjuvants to achieve the desired immune effect. For large-scale vaccination, multiple immunizations are time-consuming and laborious, increasing costs. In the current study, multiple doses (2–3 doses) are still required for BLPs vaccines to activate an effective immune response. Moreover, some BLPs vaccines alone are not immunogenic enough and require co-administration of adjuvants, such as ISA 201 VG, poly(I:C), aluminum hydroxide, complete and incomplete Freund’s adjuvant, GEL01, etc. Screening bacterial strains to produce highly antigenic BLPs or achieving simultaneous display and delivery of antigens and molecular adjuvants may be the key to solving the problem of insufficient capacity of the BLPs display platform.

With the advantages of high mucosal delivery rate, BLPs have achieved good results in the development of intranasal vaccines. However, a study has found that the ability of oral administration of BLPs to activate the immune system is not as good as that of intranasal administration (95). How to position BLPs as an antigen oral delivery system to prevent the degradation of the loaded antigen, break the immune tolerance and improve the immune effect is an issue worth exploring. First, targeted strategy has demonstrated efficacy in animal models. The binding of appropriate epithelial cell, M cell or DC receptor targeting ligands to BLPs or surface antigens in some way is promising to enhance the effect of oral immunization. Second, mucosal adjuvants such as toxins, PPRs agonists and cytokines can be used to enhance the immunogenicity of BLPs vaccines (96). Third, the use of a coating on the particles can protect the exposed antigen from degradation in the gastric environment and achieve controlled release of the antigen, thereby inducing a stronger immune response (97).

## 9 Perspective

In the past 15 years, BLPs have proved to be an efficient and economical tool with the advantages of simple preparation, low cost and high safety, etc. With many researchers’ continuous innovation and improvement of BLPs technology, BLPs have shown great application potential in adjuvant development, protein purification, enzyme immobilization technology, etc. Especially, BLPs antigen display system provides a new strategy used for subunit vaccine development. Despite much progress, improvement of BLPs vaccine efficacy remains challenging. In future work, we believe that the breakthrough of the bottleneck on BLPs display system will largely depend on the improvement of BLPs vaccine performance.

The first breakthrough point for the challenge is multivalent and combination vaccines. So far, there is no report on the antagonistic effect of simultaneously displayed antigens on BLPs and carrier-induced epitope suppression (CIES). Successful development of multivalent vaccines provides feasibility for developing BLPs combination vaccines against different pathogens, but no studies have been reported. Considering the flexibility of the combination between BLPs and antigens, antigens of different pathogens can be displayed on the same BLP simultaneously. Another format is to mix multiple BLPs displaying a single antigen. In either case, antigen dose can be controlled to achieve the maximum immune effect by adjusting to the appropriate ratio. This is undoubtedly a feasible idea to improve the performance of BLPs-based vaccine, and a critical direction to guide the research and development of BLPs vaccine.

Another point of penetration is related delivery of a wider variety of molecules on BLPs. Studies have shown that the co-presentation of adjuvant and antigen (in combination) has a better immune stimulation effect than vaccine formulations in which the adjuvant and antigen are free from each other (98). For example, some molecules related to pathogenicity or targeting molecules, can be used as adjuvants and displayed on BLP at the same time as antigens. This antigen-adjuvant-BLPs carrier vaccine platform mimics the original surface ligands and physical properties of pathogens, which is expected to improve the immune effect of vaccines. Nucleic acid vaccines are susceptible to nuclease degradation and other obstacles when they enter the body cells (99). In order to solve the safety problem of viral vectors of nucleic acid, there have been developed some non-viral vectors, such as polymers, liposomes, etc. (100) We envision BLPs as safe nucleic acid carriers to contribute to the field of gene therapy, but its ability to protect nucleic acids from degradation and efficient transport to the nucleus or ribosomes needs to be evaluated. In addition, BLPs are expected to be developed in disease diagnostic reagents based on antibody binding and molecular detection. The success of oral delivery of single-chain insulin (SCI-59) analog and the intracellular domain of insulinoma-associated protein 2 (IA-2ic) has demonstrated the potential of BLPs as drug carriers (82, 101, 102). The application of BLPs in other fields is also worth looking forward to, such as diagnostic reagents, etc.

Immunoinformatics has revolutionized the rational approach to designing vaccines. Recent outbreaks of COVID-19 pandemic and monkeypox virus have focused the scientific community on the rapid development of vaccines like never before (103). Computational methods developed and improved based on experimental immunology and large data sets save the time and cost required by traditional vaccine development methods (104). In addition, informatics has also been applied in the development of vaccine adjuvants, including receptor-ligand docking studies, analysis of factors related to gene expression, safety, mechanism of action, and effective response to vaccine adjuvants (105). Therefore, immunoinformatics has great potential to break through the existing limitations of BLPs vaccines, solve the problem of subunit vaccines, and further promote the development of vaccines.

In summary, we propose that the development of multivalent and combination vaccines, the display of more functional molecules, and the application of immunoinformatics would be breakthrough points for improving the performance of BLPs vaccines, with a hope to be directions for future research. Virus-Like Particles (VLPs), much like BLPs, possess a structure and composition resembling that of natural pathogens, enabling to mimic microbial infections and activate the immune system (106). While some VLPs vaccines for humans have been licensed and commercially successful, the application of VLPs as a veterinary vaccine platform has faced challenges, primarily due to the cost of large-scale production of VLPs (106). Therefore, we hope affordable and efficient BLPs to unlock additional unexplored potentials and make their way into the market.

## 10 Conclusions

With the development of delivery techniques, various delivery platforms show wide application in biomedical field. Here, we systematically summarize the previous research on BLPs surface display system, and track the latest progress on novel BLPs and anchors, which will provide ideas for researchers to optimize the BLPs display system for solving more problems in the field of biomedicine. As mentioned in this review, several strengths of BLPs exist, including high safety, great stability, high loading capacity and high mucosal delivery efficiency. The BLPs features allow the high potential in vaccine adjuvant, antigen carrier, antigen purification and enzyme immobilization. Overall, the research results summarized in this review have fully demonstrated that BLPs is safe and effective, easy to prepare, and has outstanding advantages and strong potential, is a promising new platform. However, improving the performance of BLPs is still challenging and further studies are needed. Improving the efficacy of vaccines based on the BLPs display system and realizing the display of more molecules on BLPs will be the future research directions, thereby opening up new avenues in molecular delivery field. We hope that this review will offer significant insights and foster additional research in field of BLPs.

## Author contributions

XZ: Resources, Writing – original draft. MG: Writing – original draft, Writing – review & editing. XD: Investigation, Writing – original draft. TS: Writing – original draft. ZB: Supervision, Writing – review & editing. JL: Supervision, Writing – review & editing. FW: Supervision, Writing – review & editing. JG: Conceptualization, Writing – review & editing.
