# Supplementary material for: Bacterium-like particles derived from probiotics: progress, challenges and prospects
Source: Front Immunol. 2023 Oct 6;14:1263586. doi: 10.3389/fimmu.2023.1263586 (PMC10587609; doi:10.3389/fimmu.2023.1263586)
Supplement: Supplementary file 1 [file DataSheet_1.docx]

Supplementary Material

**Supplementary Text 1.** The protocol for preparation of BLPs surface display system.

1) Plasmid construction and production of Protein-PA

A recombinant plasmid containing the target gene fragment and PA gene (GenBank: U17696.1, 820–1488 nt) is usually constructed to obtain recombinant protein. Proteins are commonly produced using *L. lactis*, *Escherichia coli* and baculovirus-insect cell expression system. The specific methods have been described previously(1-3).

2) BLPs preparation

The preparation process of BLPs refers to Bosma’s article(1). *L. lactis* of stationary-phase cultures were collected by centrifugation and washed once with 0.5 volume of phosphate-buffered saline (PBS) (58 mM Na_2_HPO_4_·2H_2_O, 17 mM Na_2_H_2_PO_4_·H_2_O, 68 mM NaCl; pH 7.2). Cells were resuspended in 0.2 volume of a 10% TCA solution and boiled for 30 min. Subsequently, the BLP particles formed in this way were washed three times with PBS and resuspended in PBS so that the average concentration was 2.5×10^10^ BLP particles/mL, as determined with a Burker-Turk hemocytometer. BLP particles were either used immediately for binding experiments or stored in 0.5-mL aliquots at −80℃ until use.

3) Binding of BLPs and Protein-PA

The process of binding of BLPs and Protein-PA refers to Bosma’s article(1). 2.5×10^9^ *L. lactis* BLP particles were incubated for 30 min at room temperature with 0.8 mL of 33-fold-concentrated cell-free culture supernatant containing Protein-PA. BLP particles with the bound Protein-PA were collected by centrifugation and washed twice in PBS. Finally, the BLP particles were resuspended in PBS and stored at −80℃ until use. The amount of bound Protein-PA was estimated by using Coomassie brilliant blue-stained gels and by comparison to BSA protein standards.

**Supplementary Table 1.** Immune responses elicited by different BLPs-based vaccines against viruses, bacteria, parasites and therapeutic vaccines based BLPs.

|  | Pathogen | Antigen | LAB | Bound or mixed | Animal model | Vaccination route | Additional adjuvant | Humoral response | Cellular response | Protection challenge | Cytokine response and other results |
| --- | --- | --- | --- | --- | --- | --- | --- | --- | --- | --- | --- |
| Virus | HIV-1 | gp120-MTQ | *L. lactis* | B/M | Mouse, guinea pig | i.m. | / | Serum IgG titers of 10^5^  High ID50 titers(4) | / | / | / |
|  |  |  |  |  |  | i.n. | / | Serum IgG titers of 10^3^–10^4^  sIgA in nasal, rectal and vaginal samples of mice (bound)  Low ID50 titers  Neutralizing activity of nasal washes against HIV-1 tier 1 pseudoviruses(4) | / | / | / |
|  |  |  |  | B | Rhesus macaque | i.m. and i.n. | / | Increased IgG and IgA levels in mucosal samples  Increased serum IgG (both IgG1 and IgG2) titers  NAbs against the heterologous tier 1 and tier 2 viruses | IL-2-, TNF-α- and IFN-γ-producing CD4^+^ T cells were undetectable among PBMCs | / | A balanced Th1/Th2 phenotype(5) |
|  | Influenza virus | HA, M2e | *L. lactis* | B | Mouse | i.n. | / | Increased IgG titers | / | Decreased lung viral load(6) | / |
|  |  | tHA^H5N6^-mCor1, tHA^H9N2^-mCor1 | *L. lactis* | B | Mouse, chicken | i.p. (mice)/i.m. (chicken) | (Freund's complete adjuvant) | High specific antibody titers even without an adjuvant (mice)  High HI titers (chicken)(7) | / | / | / |
|  |  | Subunit vaccine  H3N2 | *L. lactis* | M | Mouse | i.m. | / | Serum HI titers > 40  Increased specific IgG titers  IgG1 > IgG2a | / | / | Less production of IL-4  IFN-γ/IL-4 >1  Improvement in the Th1/Th2 balance(8) |
|  |  | Subunit vaccine  H3N2 | *L. lactis* | M | Mouse | i.n. | / | HI titers of ^2^log7–^2^log8  Serum IgG titers > 10^4^  High levels of serum IgA titers and sIgA titers in nasal and lung lavages  Increased IgG2a and IgG2b levels but decreased IgG1 levels | Lower numbers of IL-4-producing cells but substantially higher numbers of IFN-γ-producing cells | / | A Th1-skewed response(9) |
|  |  | Subunit vaccine  H1N1 | *L. lactis* | M | Mouse | i.n. | CTA1-GEM | High IgG and IgA response  Increased IgG1 and IgG2a responses, especially IgG2a levels | / | / | High IFN-γ levels in splenocytes  A shift of the immune response from a balanced Th1/Th2 to a predominant Th1-type response(10) |
|  |  | Subunit vaccine  H3N2 | *L. lactis* | M | Mouse | Oral/rectal | / | High serum HI titers (oral)  Serum IgG titers > 40 (oral)  sIgA titers in intestinal and nasal washes  IgG1 > IgG2 | / | / | A shift from Th2 to Th1/Th2 balance (oral)(11) |
|  |  | Split vaccine  H1N1 | *L. lactis* | M | Mouse | i.n. | / | Increased serum IgG and HI levels  Increased sIgA titers in lung, nose, and vaginal | / | 100% protected against homologous and heterologous infection  Reduction of viral titer in lungs(12) | / |
|  |  | Split vaccine  H3N2, H1N1 | *L. lactis* | M | Mouse | i.n. | / | Higher serum IgG response and sIgA levels in the nasal and lung lavages in wt mice compared with those in TLR2KO mice and mice immunized with vaccine alone  Increased IgG1 in TLR2KO mice | Lower numbers of IFN-γ producing T-cells and B-cells both in the local dLN and spleen in TLR2KO mice compared with numbers of those cells in wt control mice, while higher numbers of those cells in the local dLN in mice immunized with BLP vaccine compared with those cells in mice immunized with vaccine alone | / | Increased production of IL-17 and significantly decreased secretion of IL-5  Th1/Th17 skewing(13) |
|  |  | Inactivated vaccine  NIBRG-23 | *L. lactis* | M | Chicken | Active intratracheal route | / | Considerable IgY titers in serum and lung lavages  Increased HI titers  Increased lung IgA titers(14) | / | / | / |
|  |  |  |  |  |  | Passive inhalation | (Advax) | Increased serum IgY titers and HI titers | / | No virus either in choanal or cloacal swabs(14) | / |
|  | RSV | F | *L. lactis* | B | Mouse, cotton rat | i.n. | / | High IgG levels (15–24 fold higher than others)  Increased IgG2a/IgG1 ratio  sIgA titers in the nose | / | High specific IgG levels and virus neutralization titers  Significant reduction in lung titers  No scores of interstitial pneumonia and alveolitis | Improvement in the Th1/Th2 balance (15) |
|  | Zika virus | prM-E | *L. lactis* | B | Mouse | i.m. | ISA 201 VG, poly(I:C) | Mean PRNT50 titers > 10 | CD19^+^ CD40^+^ double-positive cells nodes and CD11_c+_ MHC II^+^ cells in the inguinal lymph  High proportions of CD4^+^ CD69^+^ T, CD8^+^ CD69^+^ T, CD19^+^ CD69^+^ B cells, CD44^+^ CD62L^+^ CD4^+^ and CD44^+^ CD62L^+^ CD8^+^ among splenic lymphocytes | / | The levels of IFN-γ, IL-4, IL-6 and IL-10 increased to varying degrees  IgG2a/IgG1 < 1(16) |
|  | Sudan virus | eGP | *L. lactis* | B | Mouse | i.m. | ISA 201VG, Poly(I:C) | High specific IgG and neutralizing antibody titers, endpoint titers up to 1:40,960 | / | / | High quantities of IL-2, IL-4, IL-10, IFN-γ and TNF-α secreted by splenocytes(17) |
|  | Classical swine fever virus | E2-Spy | *L. lactis* | B | Mouse | s.c. | ISA 206 | PI values of serum up to 92.21%  High neutralizing antibody titers(18) | / | / | / |
|  |  | E2 | *L. lactis* | B | Mouse | Inject | / | High levels of specific serum IgG with 83.112% blocking rate  High neutralizing antibody titers | / | / | Production of TNF-α, IL-6 and IL-12 in APCs(19) |
|  | PCV2 | Cap | *L. lactis* | B | Piglet | i.m. | Aluminum hydroxide | A maximum serum IgG titer of 1:3200 | / | Rectal temperature ≤ 40℃  Significantly high RIDG  No significant change for HLNs  Significant reduction in gross lung lesion scores and IHC PCV2-positive signals(20) | / |
|  | MERS-CoV | RBD | *L. lactis* | B | Mouse | i.n. | GEL01 | High levels of specific serum IgG and mucosal IgA in lung lavage fluid and intestine washes | Increased expression of CD69 on B cells, CD4^+^ T cells CD4^+^ T cells | / | High levels of IFN-γ, TNF-α, IL-2, IL-4, IL-6, and IL-10  A Th1-polarized immune response(3) |
|  | Iridoviruses | Cor1-MCP | *L. lactis* | B | Mouse | i.p. | Complete and incomplete Freund’s adjuvant | Production of specific IgG antibodies(21) | / | / | / |
|  | Rift Valley fever virus | Gn head | *L. lactis* | B | Mouse | i.m. | / | High specific IgG titers  IgG2a/IgG1< 1  Detectable IgG3  Inhibition of 50% RVFV pseudovirus infection | Elevated number of CD3^+^CD4^+^ and CD3^+^CD8^+^ T cells | / | IFN-γ and IL-4 responses[18] |
|  | HSV-2 | gD2 | */* | B | Mouse | i.n. | / | Antigen-specific IgG and sIgA production | Similar IFN-γ+ T cell frequencies with gD2 (i.n.) | 87.5% protection which was a 25% improvement compared to gD2 alone | High levels of IL-2, IFN-γ, IL-10 and IL-17A, especially IL-4  Not alter the Th1/Th2 polarization  Strong Th17 response(22) |
|  | TBEV | E protein | *L. lactis* | B | Mouse | i.m. | ISA 201VG, Poly(I:C) | robust and persistent (6 months after the second immunization) IgG antibodies  IgG2a/IgG1< 1 | Significantly higher splenocyte proliferation index  High proportions of CD11c^+^ CD80^+^, CD11c^+^ MHC I^+^, CD11c^+^ MHC II^+^, CD19^+^CD40^+^, CD19^+^CD69^+^, CD4^+^CD69^+^ and CD8^+^CD69^+^ double-positive cells  Production of double-positive cells and central memory T cells and effector memory T cells | / | Significantly high levels of IL-12p70, TNF-α, IL-4, IL-5, IL-6, IL-10 and IL-1β  A Th2-biased response(23) |
|  | Newcastle disease virus | Inactivated NDV | *L. lactis* | M | Chicken | i.n. | / | High levels of specific serum IgG and mucosal IgA  Serum HI titers up to 2^8^ | / | 100% protection | High NO concentrations and high expression of IL-1β, IL-6, IFN-γ, and iNOS(24) |
|  | Rotavirus | Live vaccine | *L.rhamnosus* | M | Mouse | Oral | / | High levels of serum IgG and intestinal IgA | Significantly increased CD3^+^CD4^+^ T cells and CD24^+^B220^+^ B cells in Peyer’s patches | / | Significantly increased production of TNF-α, IFN-γ, and IL-4 in both Peyer’s patches and spleens(25) |
|  | Hepatitis E virus | ORF2 | *L. rhamnosus* | M | Mouse | Oral | / | High levels of serum IgG and intestinal IgA | Increased CD45^+^CD3^+^CD4^+^ T cells and CD24^+^B220^+^ cells in Peyer’s patches and spleen | / | Increased levels of IFN-γ, TNF-α, and IL-4 in Peyer’s patches and spleen(26) |
| Bacteria | *Streptococcus pneumoniae* | IgA1p or/and SlrA or/and PpmA | *L. lactis* | B | Mouse | i.n. | / | Specific serum IgG | / | 70% protection (trivalent and SlrA-IgA1p divalent vaccine)  40%-50% protection (other divalent vaccines)  Reduction of loss of weight and bacterial counts in lungs, blood and nose(27) | / |
|  |  | Plym2 | *L. lactis* | B | Mouse | i.n. | / | High levels of serum IgG and sIgA in lung lavages  High HI titers(28) | / | / | / |
|  |  | PspA | *L. lactis* | B | Mouse | i.n. | / | Specific IgG in sera and sIgA in bronchoalveolar lavages of the lung and washes of the nasal passage | / | Reduction of loss of weight  100% protection against homologous and heterologous pneumococcal strain better than PPV23(29) | / |
|  |  | PspA2/PspA4 | *L. lactis* | B | Mouse | i.n. | / | High levels of specific serum IgG and specific sIgA in bronchoalveolar washes | / | 100% protection better than PPV23(30) | / |
|  | *Yersinia pestis* | LcrV | *L. lactis* | B | Mouse | i.n. | / | High levels of specific serum IgG  High IgG2a/IgG1 ratio  Increased levels of specific IgM  High numbers of IgA and IgG ASCs in the NALT | Increased numbers of IgG ASCs in the spleen, BM and NALT and IgA ASCs in NALT  Remarkable proliferation of CD4^+^ and CD8^+^ T cell especially IFN-γ-secreting CD4^+^ T cells | 100% protection | Increased levels of IL-2, IL-12, IL-6, TNF-α, and IL-10 in the NALT  Increased levels of IL-2, IL-6, IL-5 and IFN-γ in lung cells  Th1-type responses(31) |
|  | *Shigella spp.* | IpaB/IpaD | *L. lactis* | B | Mouse | i.n. | / | Significant levels of specific serum IgG especially IgG2a and IgA in stool  Specific IgG in BALf | IgA and IgG ASCs in NALT and lung tissue  High opsonophagocytic activity (OPA) responses  High capacity to neutralize macrophage cytotoxicity  Specific IgG and IgA ASCs in the spleen and BM and 40% of the BM recipients survived the pulmonary challenge | 90% protection against *S. flexneri* and 80% cross-protection against *S. sonnei* in adults  90% protection against *S. flexneri* and 40% cross-protection against *S. sonnei* in newborns | High levels of IL-1β, IL-12 and KC in the BALf Improvement in the Th1/Th2 balance(32) |
|  | *Campylobacter jejuni* | CjaA, CjaD/ rCjaAD | *L. salivarius* | B | Chicken | p.o. (CjaA, CjaD)/s.c. (CjaA, CjaD)/in ovo (rCjaAD) | / | Specific intestinal IgA (*in ovo*) | / | No protected effect i.n. or s.c. inoculation against intestinal tract colonization of wild type *C. jejuni* strain while administered *in ovo* the mean level was reduced 100 times and correlate of significant levels of protection(33) | / |
|  | *Helicobacter pylori* | CUE | *L. lactis* | B | Mouse | i.g. | / | High levels of urease-specific IgG and IgA in serum  High levels of urease-specific IgG2a and IgG1  Significantly high sIgA production in gastric tissue, duodenal mucus, caecum and faeces | High frequencies of CD4^+^ T cells in marginal zone of spleen  Specific IFN-γ T cell response | Reduction of the urease activity, gastric inflammation level and bacterial colonization | Increased IFN-γ, IL-4, IL-6 and IL-17 levels in the serum and IFN-γ levels in gastric tissue after i.n. challenged(2) |
|  |  |  |  |  |  | i.p./p.o. | / | Increased gastric sIgA (p.o.)  Increased serum specific IgG (i.p.better than p.o.)  Similar levels of IgG2a and IgG1  Increased serum IgA and IgM | Proliferation of Th17 cells in the stomach and MLN  Proliferation of CD45^+^ cells, CD3^+^ cells, CD4^+^ cells, and neutrophils (Ly6G^+^) in gastric tissue  Mild inflammation with abundant infiltration of T cells in the gastric tissue (p.o.) | Reduction of bacterial colonization (p.o.) in gastric tissue  Increased S100A8 and MUC1 expressions (p.o.) | Increased levels of IFN-γ, IL-6 and IL-7 in spleens cells and IFN-γ, IL-17, CXCL1 and CXCL2 in gastric tissue  Th1/Th17 memory response(34) |
|  | *Klebsiella pneumoniae* | OmpK17 or/and  OmpK36 | *L. lactis* | M | Mouse | s.c. | / | No significant levels of specific IgG1 or IgG2a antibodies | No significant enhancement of phagocytic activity of PMN cells | No significant protection(35) | / |
|  | *Bordetella pertussis* | PT, FHA and PRN | *L. lactis* | M | Mouse | i.n. | / | Remarkable serum IgG antibodies  Mucosal anti-PT IgA and anti-FHA IgA in nasal washes | / | Reduced bacterial load in the lungs  No notable histopathological damage in the lungs(36) | / |
| Parasites | *Plasmodium falciparum* | MSA2 | *L. lactis* | B | Rabbit | p.o. | / | Serum antibody titers reach 10^-4^ | / | / | Reduced anti-carrier response(37) |
|  | *Plasmodium berghei* | CSP | *L. lactis* | B | Mouse | s.c. | / | High IgG antibody levels | High CTL- and Th-specific IFN-γ responses in splenocytes | 100% protection(38) | / |
| Others | Alzheimer’s disease | Amyloid-β | *L. lactis* | B | Mouse | i.m. | / | High levels of specific antibody with excellent persistence (3 mouths) | Blockade of in vitro cytotoxicity (90% protection)(39) | / | / |
|  | Type 1 diabetes mellitus | SCI-59 | *L. lactis* | B | Mouse | p.o. | / | High serum IgG especially IgG1 levels but no significant difference among various groups for IgG2a | Suppression of splenocyte proliferation  High content of CD4^+^CD25^+^FoxP3^+^ Tregs in the PLN CD4^+^ T cell compartment | / | Low secretion of Th1-type cytokines (IL-2 and IFN-γ)  Increased Th2-type cytokines (IL-4 and IL-10) production  Significant reduction in diabetes incidence and insulitis  Short maintenance time of hyperglycemia in OGTT  Superior maintenance of C-peptide secretion  Th2-type humoral immune responses (Repair of Th1/Th2 imbalance)(40) |
|  |  | IA-2ic | *L. lactis* | B | Mouse | p.o. | / | same as above | same as above | / | same as above(41) |

**Supplementary Material References**

1. Bosma T, Kanninga R, Neef J, Audouy SAL, van Roosmalen ML, Steen A, et al. Novel surface display system for proteins on non-genetically modified gram-positive bacteria. *Appl Environ Microbiol* (2006) 72(1):880-9.

2. Liu W, Tan Z, Xue J, Luo W, Song H, Lv X, et al. Therapeutic efficacy of oral immunization with a non-genetically modified Lactococcus lactis-based vaccine CUE-GEM induces local immunity against Helicobacter pylori infection. *Appl Microbiol Biotechnol* (2016) 100(14):6219-29. doi: 10.1007/s00253-016-7333-y.

3. Li E, Chi H, Huang P, Yan F, Zhang Y, Liu C, et al. A Novel Bacterium-Like Particle Vaccine Displaying the MERS-CoV Receptor-Binding Domain Induces Specific Mucosal and Systemic Immune Responses in Mice. *Viruses* (2019) 11(9). doi: 10.3390/v11090799.

4. Bi J, Li F, Zhang M, Wang H, Lu J, Zhang Y, et al. An HIV-1 vaccine based on bacterium-like particles elicits Env-specific mucosal immune responses. *Immunol Lett* (2020) 222:29-39. doi: 10.1016/j.imlet.2020.03.002.

5. Wang H, Li P, Zhang M, Bi J, He Y, Li F, et al. Vaccine with bacterium-like particles displaying HIV-1 gp120 trimer elicits specific mucosal responses and neutralizing antibodies in rhesus macaques. *Microb Biotechnol* (2022) 15(7):2022-39. doi: 10.1111/1751-7915.14022.

6. Van Braeckel-Budimir N, Haijema BJ, Leenhouts K. Bacterium-like particles for efficient immune stimulation of existing vaccines and new subunit vaccines in mucosal applications. *Front Immunol* (2013) 4:282. doi: 10.3389/fimmu.2013.00282.

7. Song S-J, Shin G-I, Noh J, Lee J, Kim D-H, Ryu G, et al. Plant-based, adjuvant-free, potent multivalent vaccines for avian influenza virus via Lactococcus surface display. *J Integr Plant Biol* (2021) 63(8):1505-20. doi: 10.1111/jipb.13141.

8. Saluja V, Visser MR, Ter Veer W, van Roosmalen ML, Leenhouts K, Hinrichs WLJ, et al. Influenza antigen-sparing by immune stimulation with Gram-positive enhancer matrix (GEM) particles. *Vaccine* (2010) 28(50):7963-9. doi: 10.1016/j.vaccine.2010.09.066.

9. Saluja V, Amorij JP, van Roosmalen ML, Leenhouts K, Huckriede A, Hinrichs WLJ, et al. Intranasal delivery of influenza subunit vaccine formulated with GEM particles as an adjuvant. *AAPS J* (2010) 12(2):109-16. doi: 10.1208/s12248-009-9168-2.

10. Zhang Y, Yu X, Hou L, Chen J, Li P, Qiao X, et al. CTA1: Purified and display onto gram-positive enhancer matrix (GEM) particles as mucosal adjuvant. *Protein Expr Purif* (2018) 141:19-24. doi: 10.1016/j.pep.2017.08.010.

11. Saluja V, Visser MR, van Roosmalen ML, Leenhouts K, Huckriede A, Hinrichs WLJ, et al. Gastro-intestinal delivery of influenza subunit vaccine formulation adjuvanted with Gram-positive enhancer matrix (GEM) particles. *Eur J Pharm Biopharm* (2010) 76(3):470-4. doi: 10.1016/j.ejpb.2010.08.003.

12. de Haan A, Haijema BJ, Voorn P, Meijerhof T, van Roosmalen ML, Leenhouts K. Bacterium-like particles supplemented with inactivated influenza antigen induce cross-protective influenza-specific antibody responses through intranasal administration. *Vaccine* (2012) 30(32):4884-91. doi: 10.1016/j.vaccine.2012.04.032.

13. Keijzer C, Haijema BJ, Meijerhof T, Voorn P, de Haan A, Leenhouts K, et al. Inactivated influenza vaccine adjuvanted with bacterium-like particles induce systemic and mucosal influenza A virus specific T-cell and B-cell responses after nasal administration in a TLR2 dependent fashion. *Vaccine* (2014) 32(24):2904-10. doi: 10.1016/j.vaccine.2014.02.019.

14. Tomar J, Biel C, de Haan CAM, Rottier PJM, Petrovsky N, Frijlink HW, et al. Passive inhalation of dry powder influenza vaccine formulations completely protects chickens against H5N1 lethal viral challenge. *Eur J Pharm Biopharm* (2018) 133:85-95. doi: 10.1016/j.ejpb.2018.10.008.

15. Rigter A, Widjaja I, Versantvoort H, Coenjaerts FEJ, van Roosmalen M, Leenhouts K, et al. A protective and safe intranasal RSV vaccine based on a recombinant prefusion-like form of the F protein bound to bacterium-like particles. *PLoS One* (2013) 8(8):e71072. doi: 10.1371/journal.pone.0071072.

16. Jin H, Bai Y, Wang J, Jiao C, Liu D, Zhang M, et al. A bacterium-like particle vaccine displaying Zika virus prM-E induces systemic immune responses in mice. (2021).

17. Xu S, Jiao C, Jin H, Li W, Li E, Cao Z, et al. A Novel Bacterium-Like Particle-Based Vaccine Displaying the SUDV Glycoprotein Induces Potent Humoral and Cellular Immune Responses in Mice. *Viruses* (2019) 11(12). doi: 10.3390/v11121149.

18. Li D, Zhang H, Yang L, Chen J, Zhang Y, Yu X, et al. Surface display of classical swine fever virus E2 glycoprotein on gram-positive enhancer matrix (GEM) particles via the SpyTag/SpyCatcher system. *Protein Expr Purif* (2020) 167:105526. doi: 10.1016/j.pep.2019.105526.

19. Hu M, Wang F, Li N, Xing G, Sun X, Zhang Y, et al. An antigen display system of GEM nanoparticles based on affinity peptide ligands. *Int J Biol Macromol* (2021) 193(Pt A):574-84. doi: 10.1016/j.ijbiomac.2021.10.135.

20. Li P-C, Qiao X-W, Zheng Q-S, Hou J-B. Immunogenicity and immunoprotection of porcine circovirus type 2 (PCV2) Cap protein displayed by Lactococcus lactis. *Vaccine* (2016) 34(5):696-702. doi: 10.1016/j.vaccine.2015.09.007.

21. Ahn G, Cha J-Y, Lee JW, Park G, Shin G-I, Song S-J, et al. Production of a Bacteria-like Particle Vaccine Targeting Rock Bream (Oplegnathus fasciatus) Iridovirus Using Nicotiana benthamiana. *J Plant Biol* (2022) 65(1):21-8. doi: 10.1007/s12374-021-09328-z.

22. Wan M, Yang X, Chen Z, Su W, Cai L, Hou A, et al. Comparison of Effects of Multiple Adjuvants and Immunization Routes on the Immunogenicity and Protection of HSV-2 gD Subunit Vaccine. *Immunol Lett* (2023). doi: 10.1016/j.imlet.2023.06.004.

23. Zhang M, Jin H, Li Y, Jiao C, Huang P, Bai Y, et al. Genetically engineered bacterial-like particles induced specific cellular and humoral immunity as effective tick-borne encephalitis virus vaccine. n/a(n/a):e305. doi: https://doi.org/10.1002/agt2.305.

24. Yang R, Zhang S, Yu Y, Hong X, Wang D, Jiang Y, et al. Adjuvant effects of bacterium-like particles in the intranasal vaccination of chickens against Newcastle disease. *Vet Microbiol* (2021) 259:109144. doi: 10.1016/j.vetmic.2021.109144.

25. Raya Tonetti F, Arce L, Salva S, Alvarez S, Takahashi H, Kitazawa H, et al. Immunomodulatory Properties of Bacterium-Like Particles Obtained From Immunobiotic Lactobacilli: Prospects for Their Use as Mucosal Adjuvants. *Front Immunol* (2020) 11:15. doi: 10.3389/fimmu.2020.00015.

26. Arce LP, Raya Tonetti MF, Raimondo MP, Müller MF, Salva S, Álvarez S, et al. Oral Vaccination with Hepatitis E Virus Capsid Protein and Immunobiotic Bacterium-Like Particles Induce Intestinal and Systemic Immunity in Mice. *Probiotics Antimicrob Proteins* (2020) 12(3):961-72. doi: 10.1007/s12602-019-09598-7.

27. Audouy SAL, van Selm S, van Roosmalen ML, Post E, Kanninga R, Neef J, et al. Development of lactococcal GEM-based pneumococcal vaccines. *Vaccine* (2007) 25(13):2497-506.

28. Lu J, Hou H, Wang D, Leenhouts K, Roosmalen MLv, Sun T, et al. Systemic and mucosal immune responses elicited by intranasal immunization with a pneumococcal bacterium-like particle-based vaccine displaying pneumolysin mutant Plym2. *Immunol Lett* (2017) 187:41-6. doi: 10.1016/j.imlet.2017.05.003.

29. Wang D, Lu J, Yu J, Hou H, Leenhouts K, Van Roosmalen ML, et al. A Novel PspA Protein Vaccine Intranasal Delivered by Bacterium-Like Particles Provides Broad Protection Against Pneumococcal Pneumonia in Mice. *Immunol Invest* (2018) 47(4):403-15. doi: 10.1080/08820139.2018.1439505.

30. Lu J, Guo J, Wang D, Yu J, Gu T, Jiang C, et al. Broad protective immune responses elicited by bacterium-like particle-based intranasal pneumococcal particle vaccine displaying PspA2 and PspA4 fragments. *Hum Vaccin Immunother* (2019) 15(2):371-80. doi: 10.1080/21645515.2018.1526556.

31. Ramirez K, Ditamo Y, Rodriguez L, Picking WL, van Roosmalen ML, Leenhouts K, et al. Neonatal mucosal immunization with a non-living, non-genetically modified Lactococcus lactis vaccine carrier induces systemic and local Th1-type immunity and protects against lethal bacterial infection. *Mucosal Immunol* (2010) 3(2):159-71. doi: 10.1038/mi.2009.131.

32. Heine SJ, Franco-Mahecha OL, Chen X, Choudhari S, Blackwelder WC, van Roosmalen ML, et al. Shigella IpaB and IpaD displayed on L. lactis bacterium-like particles induce protective immunity in adult and infant mice. *Immunol Cell Biol* (2015) 93(7):641-52. doi: 10.1038/icb.2015.24.

33. Kobierecka PA, Wyszyńska AK, Gubernator J, Kuczkowski M, Wiśniewski O, Maruszewska M, et al. Chicken Anti-Campylobacter Vaccine - Comparison of Various Carriers and Routes of Immunization. *Front Microbiol* (2016) 7:740. doi: 10.3389/fmicb.2016.00740.

34. Liu W, Tan Z, Liu H, Zeng Z, Luo S, Yang H, et al. Nongenetically modified Lactococcus lactis-adjuvanted vaccination enhanced innate immunity against Helicobacter pylori. *Helicobacter* (2017) 22(5). doi: 10.1111/hel.12426.

35. Hussein KE, Bahey-El-Din M, Sheweita SA. Immunization with the outer membrane proteins OmpK17 and OmpK36 elicits protection against Klebsiella pneumoniae in the murine infection model. *Microb Pathog* (2018) 119:12-8. doi: 10.1016/j.micpath.2018.04.004.

36. Shi W, Kou Y, Jiang H, Gao F, Kong W, Su W, et al. Novel intranasal pertussis vaccine based on bacterium-like particles as a mucosal adjuvant. *Immunol Lett* (2018) 198:26-32. doi: 10.1016/j.imlet.2018.03.012.

37. Ramasamy R, Yasawardena S, Zomer A, Venema G, Kok J, Leenhouts K. Immunogenicity of a malaria parasite antigen displayed by Lactococcus lactis in oral immunisations. *Vaccine* (2006) 24(18):3900-8.

38. Nganou-Makamdop K, van Roosmalen ML, Audouy SAL, van Gemert G-J, Leenhouts K, Hermsen CC, et al. Bacterium-like particles as multi-epitope delivery platform for Plasmodium berghei circumsporozoite protein induce complete protection against malaria in mice. *Malar J* (2012) 11:50. doi: 10.1186/1475-2875-11-50.

39. Fu L, Guo Y, Sun Y, Dong Y, Wu J, Yu B, et al. A novel Aβ epitope vaccine based on bacterium-like particle against Alzheimer's disease. *Mol Immunol* (2018) 101:259-67. doi: 10.1016/j.molimm.2018.07.019.

40. Mao R, Chen Y, Wu Q, Zhang T, Diao E, Wu D, et al. Oral delivery of single-chain insulin (SCI-59) analog by bacterium-like particles (BLPs) induces oral tolerance and prevents autoimmune diabetes in NOD mice. *Immunol Lett* (2019) 214:37-44. doi: 10.1016/j.imlet.2019.08.008.

41. Mao R, Yang M, Yang R, Chen Y, Diao E, Zhang T, et al. Oral delivery of the intracellular domain of the insulinoma-associated protein 2 (IA-2ic) by bacterium-like particles (BLPs) prevents type 1 diabetes mellitus in NOD mice. *Drug Deliv* (2022) 29(1):925-36. doi: 10.1080/10717544.2022.2053760.
